# The intranasal dexmedetomidine plus ketamine for procedural sedation in children, adaptive randomized controlled non-inferiority multicenter trial (Ketodex): a statistical analysis plan

**DOI:** 10.1186/s13063-020-04946-3

**Published:** 2021-01-06

**Authors:** Anna Heath, Juan David Rios, Eleanor Pullenayegum, Petros Pechlivanoglou, Martin Offringa, Maryna Yaskina, Rick Watts, Shana Rimmer, Terry P. Klassen, Kamary Coriolano, Naveen Poonai, on behalf of the PERC-KIDSCAN Ketodex Study Group, Darcy Beer, Scott Sawyer, Maala Bhatt, April Kam, Quynh Doan, Vikram Sabhaney, Serena Hickes, Samina Ali, Karly Stillwell, Tannis Erickson, Chelsea Bowkett, Carolyn Shimmin, Brendon Foot, Chelsea Bowkett, Candace McGahern, Redjana Carciurmaruj, Jeannine Schellenberg

**Affiliations:** 1 Child Health Evaluative Sciences, The Hospital for Sick Children, Toronto, Canada; 2 Dalla Lana School of Public Health, Division of Biostatistics, University of Toronto, Toronto, Canada; 3 Department of Statistical Science, University College London, London, UK; 4 Institute of Health Policy, Management and Evaluation, University of Toronto, Toronto, Ontario Canada; 5 Division of Neonatology, The Hospital for Sick Children, University of Toronto, Toronto, Ontario Canada; 6 Women & Children’s Health Research Institute, University of Alberta, Edmonton, Alberta Canada; 7 University of Manitoba, Winnipeg, Manitoba Canada; 8 Children’s Hospital Research Institute of Manitoba, Winnipeg, Manitoba Canada; 9 London Health Sciences Centre, Children’s Hospital, London, Ontario Canada; 10 Departments of Paediatrics and Epidemiology & Biostatistics, Schulich School of Medicine and Dentistry, London, Canada; 11 Children’s Health Research Institute, London Health Sciences Centre, London, Canada

**Keywords:** Procedural sedation and analgesia, Pediatric closed reduction, Intranasal ketodex, Non-inferiority trial, Bayesian adaptive design, Statistical analysis plan

## Abstract

**Background:**

Procedural sedation and analgesia (PSA) is frequently required to perform closed reductions for fractures and dislocations in children. Intravenous (IV) ketamine is the most commonly used sedative agent for closed reductions. However, as children find IV insertion a distressing and painful procedure, there is need to identify a feasible alternative route of administration. There is evidence that a combination of dexmedetomidine and ketamine (ketodex), administered intranasally (IN), could provide adequate sedation for closed reductions while avoiding the need for IV insertion. However, there is uncertainty about the optimal combination dose for the two agents and whether it can provide adequate sedation for closed reductions. The Intranasal Dexmedetomidine Plus Ketamine for Procedural Sedation (Ketodex) study is a Bayesian phase II/III, non-inferiority trial in children undergoing PSA for closed reductions that aims to address both these research questions. This article presents in detail the statistical analysis plan for the Ketodex trial and was submitted before the outcomes of the trial were available for analysis.

**Methods/design:**

The Ketodex trial is a multicenter, four-armed, randomized, double-dummy controlled, Bayesian response adaptive dose finding, non-inferiority, phase II/III trial designed to determine (i) whether IN ketodex is non-inferior to IV ketamine for adequate sedation in children undergoing a closed reduction of a fracture or dislocation in a pediatric emergency department and (ii) the combination dose for IN ketodex that provides optimal sedation. Adequate sedation will be primarily measured using the Pediatric Sedation State Scale. As secondary outcomes, the Ketodex trial will compare the length of stay in the emergency department, time to wakening, and adverse events between study arms.

**Discussion:**

The Ketodex trial will provide evidence on the optimal dose for, and effectiveness of, IN ketodex as an alternative to IV ketamine providing sedation for patients undergoing a closed reduction. The data from the Ketodex trial will be analyzed from a Bayesian perspective according to this statistical analysis plan. This will reduce the risk of producing data-driven results introducing bias in our reported outcomes.

**Trial registration:**

ClinicalTrials.gov NCT04195256. Registered on December 11, 2019.

## Background

Orthopedic injuries are common in children visiting the emergency department (ED) [1, 2], sometimes requiring a closed reduction [3, 4], which usually requires procedural sedation and analgesia (PSA). Intravenous (IV) ketamine is a common sedative agent that facilitates closed reductions [5]. However, IV insertion is distressing for children and their caregivers and can be challenging as children have small veins and resist positioning [6].

Intranasal (IN) administration of sedative agents is a less invasive potential alternative to IV insertion [7] with a combination of dexmedetomidine and ketamine (ketodex) showing promise for PSA across several indications [8]. However, it is unclear whether IN ketodex would offer sufficient analgesia and sedation for closed reductions in children with orthopedic injuries, compared to IV ketamine, and there is no evidence on the most effective combination of dexmedetomidine and ketamine within the ketodex formulation.

The intranasal dexmedetomidine plus ketamine for procedural sedation in children (Ketodex) study aims to determine (i) whether the sedation provided by IN ketodex is non-inferior to that provided by IV ketamine for children undergoing a closed reduction and (ii) the combination dose of IN ketodex that provides optimal sedation. To achieve these objectives, the Ketodex study uses an innovative Bayesian response-adaptive, comparative-effectiveness design [9]. This paper outlines the statistical analysis plan (SAP) for the Ketodex trial as the study protocol is published separately [10]. This SAP has been published before analyzing any study data and follows the reporting guidelines for SAPs [11].

### Objectives

The primary aim of the Ketodex study is to determine if IN ketodex is non-inferior to IV ketamine with respect to the proportion of participants who achieve adequate sedation for the duration of a closed reduction. We will enroll previously healthy children who present to one of six Canadian participating pediatric EDs requiring a reduction for a fracture or dislocation. The secondary objectives of the study are to determine the optimal combination dose for IN ketodex and to characterize the sedation characteristics of IN ketodex with respect to length of stay in the ED, need for additional sedation, nasal irritation, satisfaction with sedation, and adverse events.

## Methods/design

### Design and setting

The Ketodex trial is a phase II/III, double-dummy, controlled, randomized, Bayesian response adaptive, dose finding, non-inferiority trial being conducted in six Canadian tertiary care pediatric EDs. Eligible participants are age 4 to 17 years and require PSA to undergo a closed reduction of a fracture or dislocation. Following informed consent, patients will be randomized in a 2:3 ratio to receive either IV ketamine, dosed at 1.5 mg/kg (maximum 100 mg), with an IN placebo combination treatment, or one of three combination doses of IN ketodex, i.e., 2, 3, or 4 mg/kg (maximum 400 mg) of ketamine combined with 4, 3, or 2 μg/kg (maximum 200 μg) of dexmedetomidine, respectively, with an IV placebo. Patients will then be further randomized to receive one of the three dose combinations, initially in a 1:1:1 ratio (see “Randomization”).

Most study data will be collected in the ED within 1–2 h of study enrollment. A follow-up phone call will be made between 24 and 48 h after enrollment to collect data on issues with the sedation. If a participant is lost to telephone follow-up, a research nurse will attempt to regain contact 3–5 times, using email or a letter to the participant’s last known address, depending on local research ethics board (REB) regulations.

#### Study protocol development and conduct

The Ketodex study was registered on ClinicalTrials.gov on December 11, 2019, with registration number NCT04195256. At each institution, the REB will give ethical approval before commencing local enrollment. Informed consent, following institutional REB guidelines, will be obtained from caregivers before randomization and data collection. The Ketodex study will be supported by the KidsCAN-PERC iPCT network [12], a Canadian trials network using centralized infrastructure for data management and trial oversight for four trials. An independent data and safety monitoring board (DSMB) of six oversees the study.

### Randomization

**Fig. 1:**
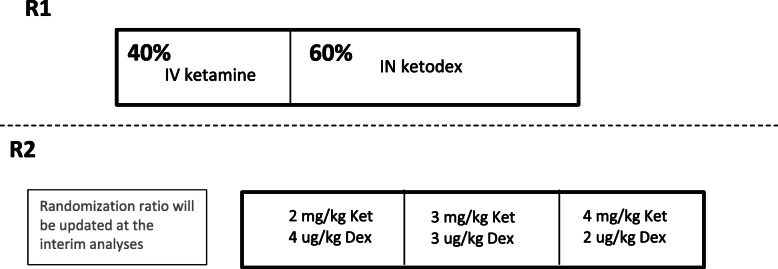
Depiction of the randomization procedure for the Ketodex trial

Participants will be randomized in a ratio of 2:3 to either IV ketamine or IN ketodex using block randomization with blocks of size five, stratified by site. Participants will undergo a further randomization to one of the three IN ketodex combinations using the REDCap electronic data capture system [13]. For participants randomized to IV ketamine, the second randomization will be to two placebo (saline) solutions. The second randomization step will be adapted so more participants receive the more effective dose combinations. Figure 1 displays this two-step randomization procedure.

The initial randomization ratio for the dose combinations will be 1:1:1. Interim analyses will update the randomization ratio at approximately 150, 200, 250, 300, and 350 participants. As outlined in the “Analysis for the primary endpoint” section, each interim analysis will compute the posterior probability that each dose is the most effective. The number of patients randomized to each dose before the next interim analysis will equal this posterior probability multiplied by the number of participants to be enrolled before the next interim analysis, rounded to the nearest whole number. If, at a given interim analysis, the probability that a dose is optimal falls below 0.05 (threshold chosen by simulation), we will not randomize participants to this dose. If all three combinations have a probability of being the most effective of less than 50% once 250 participants have been enrolled, then safety and tolerability will be assessed to determine the most promising combination. This dose combination will be used for all participants randomized to IN ketodex.

The randomization list for the first step will be generated and held securely at the Women and Children’s Health Research Institute’s (WCHRI) Data Coordinating Centre at the University of Alberta [14]. Pharmacies will prepare identically appearing IV ketamine plus IN saline or IN ketodex plus IV saline kits for use at the bedside. The second randomization will take place at the bedside from a master randomization list, accessed sequentially as participants are enrolled across all sites. Site-level stratification is not used for the second randomization. The research nurse will be blinded to the intervention but not to the dose combination. Outcome assessors will be blinded to the dose combination and the intervention.

### Data storage and collection

All participant data will be stored in REDCap [13] and held at the WCHRI Data Coordinating Centre [14]. All data will be entered into REDCap using a WiFi-enabled encrypted iPad or recorded on paper in the event of a technical failure. Prior to analysis, all personal identifiers will be removed and participants will be identified using a unique study identification number.

### Primary outcome

The primary outcome is adequate sedation for the duration of the closed reduction. This will be ascertained over the interval of time from the first application of traction or manipulation of the injured limb for the purpose of anatomical realignment to the last application of a realigning force. Adequate sedation is defined as fulfillment of all three of the following criteria:

i) A Pediatric Sedation State Scale (PSSS) [15] score of 2 or 3 for the duration of the procedure AND
ii) No additional medication given during the closed reduction for the purpose of sedation AND
iii) The patient did not actively resist, cry, or require physical restraint for completion of the closed reduction.

The primary outcome will be assessed by two independent outcome assessors blinded to the study group and objectives who will use video recordings of the closed reduction to calculate the PSSS score every 30 s during the procedure. Video scoring for each participant will be undertaken within 24–48 h of data collection. The second outcome assessor will score 25% of the participants to generate an interrater agreement.

### Secondary efficacy outcomes

The Ketodex trial will investigate three secondary outcomes:

1. Length of stay: Time (in minutes) recorded in the medical record between ED triage and ED discharge.
2. Time to wakening: This is the time interval between the first pair or IN sprays to the first PSSS score > 3 post-closed reduction.
3. Adverse events (AEs): The occurrence of any AE, recorded using the medical record and queries of the health care staff during sedation and recovery. Research nurses will be trained on the recognition and definition of expected and unexpected AEs. AEs will be based on Health Canada reporting standards.

### Additional outcomes

The Ketodex trial will record seven additional outcomes:

1. Length of stay due to PSA: Time (in minutes) from the first pair of IN sprays/IV dose to ED discharge.
2. Duration of procedure: Time (in minutes) of the first pair of IN sprays/IV dose to the end of cast or splint application (closed reduction).
3. Length of ED stay: time interval between triage assessment and discharge.
4. Caregiver, participant, bedside nurse or respiratory therapist, and physician satisfaction: Satisfaction from the caregiver and participant, measured using a 100-mm visual analog scale, obtained immediately prior to discharge. Satisfaction from each health care provider, measured using a 100-mm visual analog scale immediately following cast/splint application.
5. Nasal irritation: Discomfort associated with nasal sprays (if recalled), assessed by the research nurse using the Faces Pain Scale - Revised at discharge [16].
6. Volume of intervention: the volume of IN intervention a patient received, compared to the volume of IN intervention they were calculated to receive, recorded at the index visit.
7. Adjunctive IV therapy and medications: the requirement of an IV for therapy unrelated to sedation.
8. Patient preference for the method of sedation: We will ask the participant to choose their preferred sedation method, if they have one, at the index visit

### Sample size calculation

We used the average length criterion (ALC) for Bayesian sample size estimation [17] by selecting the smallest sample size such that the 95% high-density posterior credible interval for the difference in the probability of adequate sedation had an average length of 0.07, six times shorter than the prior 95% highest-posterior density credible interval (“Analysis for primary endpoint”). This reduction was selected based on practical constraints [17]. We also used ALC to determine the randomization ratio between IV ketamine and IN ketodex; we considered randomizing 20%, 30%, 40%, and 50% of patients to IV ketamine.

The ALC initially selected the smallest sample size for which the average length of the 95% high-density posterior credible interval fell below 0.07 and then selected the randomization ratio that led to the most balanced trial, provided the ALC remained below 0.07. We simulated 2000 samples from the prior predictive distribution of the data, across all four randomization regimes, for sample sizes increasing from 350 to 500 in increments of 10. For each simulation, we estimated the length of the 95% high-density posterior credible interval using 2000 simulations from the posterior. Based on this, the sample size for the Ketodex trial is 410 patients with a 2:3 randomization ratio between IV ketamine and IN ketodex.

We expect minimal missing data as the primary outcome is collected during a procedure that must be completed by the physician before discharge. Thus, the sample size is not adjusted for loss to follow-up. We will monitor missingness and adjust our recruitment target to ensure that 410 patients record the primary outcome.

### Interim analysis and stopping guidance

The Ketodex trial will have seven interim analyses, at increments of 50 enrolled participants. The DSMB will review safety outcomes DSMB at each interim analysis based on descriptive statistics of the safety outcomes between treatment groups. They may also receive posterior credible intervals or predictive probabilities. The decision to stop the trial for safety reasons is at the discretion of the DSMB. Due to the uneven treatment allocation, the DSMB will be unblinded to treatment assignment. The randomization ratio at the 2nd level of randomization will be updated after recruitment hits approximately 150, 200, 250, 300, and 350 participants. We will not undertake comparative effectiveness analyses at the interim analyses or stop for efficacy or futility.

### Statistical analysis plan

#### Statistical principles

The primary analysis of the primary outcome will take place after every participant has completed the protocol and all data have been collected and cleaned. The statistical analysis will be performed unblinded to participant allocation. The primary analysis will determine if the optimal IN ketodex combination is non-inferior to IV ketamine. All other analyses will test for superiority of IN ketodex. We will undertake an intention-to-treat analysis, including all randomized participants, and a per-protocol analysis, concluding non-inferiority if both these analyses confirm non-inferiority.

All analyses will use a Bayesian perspective with significance declared based on the posterior probability that the proposed hypothesis is true. No adjustments will be made for multiplicity due to the likelihood principle [18] with the thresholds for declaring significance chosen using simulations to control the type I error of the trial [9]. Specifically, if this probability is less than 3.7%, we will declare sufficient evidence *against* the hypothesis. If the probability is greater than 60.8%, we will declare significant evidence *for* the hypothesis. If this probability is between 3.7% and 60.8%, we will declare that the trial is *inconclusive*. We will report treatment effect estimates using 95% highest-density posterior credible intervals. The statistical analysis will be undertaken in R [19] as an interface to JAGS [20].

#### Handling of missing data

We anticipate minimal missing data as the majority of outcomes are collected within the ED. If the proportion of missing data is below 5%, we will undertake a complete case analysis. If the level of missingness exceeds 5%, we will use a joint Bayesian model for the missingness and the outcome.

#### Patient flow

We will present patient flow with a CONSORT 2010 flow diagram reporting the number of participants deemed eligible for the trial at screening and those excluded as they met a study exclusion criterion and the number of participants who were randomized and received the randomized allocation. Participants can withdraw from the study at any time, for any reason. The number of participants who withdraw and the number of participants lost to follow-up will be summarized by treatment arm.

#### Protocol deviations and adherence

A protocol deviation is any noncompliance with the clinical trial protocol, International Conference on Harmonization Good Clinical Practice, or Trial Manual of Procedures requirements. Any change, divergence, or departure from the study design or procedures constitutes a protocol deviation. The noncompliance may be on the part of either the participant, the participating site investigator, or the study site staff. The proportion of protocol deviations will be presented by the treatment group alongside descriptive information about the deviation. Adherence will be defined as a participant who received any of the study medications and will be presented by the treatment group.

#### Baseline characteristics

We will collect the participants’ age and sex, type of fracture or dislocation, location of fracture or dislocation, the identity of the person performing the closed reduction and, if required, the identity of the person performing the casting. Baseline data will be summarized using frequencies and percentages for categorical variables and means, medians, standard deviations, and interquartile ranges for continuous variables.

#### Analysis for the primary endpoint

##### Interim analyses

The adaptive randomization will be based on the probability of adequate sedation for each dose, denoted *p*_*i*_, *i* = 1, 2, 3. We assume a binomial likelihood for the data and a beta prior with parameters 6.25 and 0.25 for each dose. This prior is informed by previous data [21] and then down-weighted by 4 to an effective sample size of 6.5 [22]. Using the same prior for all three doses assumes they have the same effectiveness unless the data indicate otherwise. This prevents early stopping of a dose combination. We will compute the probability that each dose combination has the highest proportion of successfully sedated participants from the posterior distributions for *p*_*i*_, *i* = 1, 2, 3. This procedure has an 83% chance of randomizing the highest number of patients to the optimal treatment [9].

##### Dose response modeling

Participants who are not adequately sedated can be over-sedated (PSSS score of 0 or 1) or under-sedated (PSSS score of 4 or 5) [15]. The final analysis will use a monotonic log-logistic dose response model for the probability of over- and under-sedation [23] and a multinomial distribution to infer the probability of adequate sedation. Specifically, let ***X***_*i*_, for *i* = 1, 2, 3, be a three-vector containing the number of patients who experience under-, adequate, and over-sedation from the *N*_*i*_ patients that receive dose *i*. We model **X**_i_~Multinomial(*N*_*i*_, ***p***_***i***_), where ${\boldsymbol{p}}_{\boldsymbol{i}}=\left({p}_i^{(1)},{p}_i^{(2)},{p}_i^{(3)}\right)$ and

$$ {p}_i^{(1)}=\mathrm{logit}\left(\ {\beta}_1+{\beta}_2\log \left({K}_i\right)+{\beta}_3\log \left({D}_i\right)\right), $$

$$ {p}_i^{(3)}=\mathrm{logit}\left(\ {\beta}_a+{\beta}_b\log \left({K}_i\right)+{\beta}_c\log \left({D}_i\right)\right), $$

with *K*_*i*_ the dose of IN ketamine and *D*_*i*_ the dose of IN dexmedetomidine in the Ketodex combination. Interactions cannot be estimated accurately and are not required in dose finding studies [24]. The optimal dose combination is the dose with the highest expected probability of adequate sedation.

##### Priors for the dose response model

We will use non-central *t* distributions with precision 0.001 and degrees of freedom 1, suggested by [25]. The mean of *β*_1_ and *β*_*a*_ assume that 5% of participants are under-sedated and 2% over-sedated, setting the prior means for ${p}_i^{(2)},i=1,2,3$ to 0.93, the effectiveness from the literature [21]. The priors for *β*_2_, *β*_3_, *β*_*b*_ and *β*_*c*_ are centered on 0 so all dose combinations initially have the same effectiveness.

##### Effectiveness analysis

The primary effectiveness analysis will determine whether IN ketodex is non-inferior to IV ketamine in terms of providing adequate sedation. This analysis compares the effectiveness of IV ketamine and the optimal IN ketodex combination. The probability of adequate sedation with IV ketamine (*p*_IV_) is estimated using a binomial likelihood and a beta prior distribution with parameters 15.1 and 0.4. These prior parameters are estimated from published data [26] and down-weighted to prevent significant impact on the trial results.

The primary analysis is based on the posterior probability that IN ketodex is non-inferior to IV ketamine.

*γ* = *P*( *p*_IV_ − *p*_IN_ > *η*),

where *η* = 0.178 is the non-inferiority margin, estimated from our team’s survey of 204 ED physicians (outlined in the supplementary material), and *p*_IN_ is the probability of adequate sedation for the optimal IN ketodex combination. We will declare that IN ketodex is non-inferior to IV ketamine if *γ* ≤ 0.037. Other values of *γ* will induce alternative conclusions. This decision rule based on *γ* has a type I error of 5% when *p*_IV_ = 0.97 and *p*_IN_ = 0.792 and a type II error of 7% when *p*_IN_ = 0.9 [9].

#### Analysis for secondary endpoints

##### Secondary outcomes

For length of stay, onset of sedation, and duration of sedation, we will use a linear dose-response model to estimate the mean duration for the optimal IN ketodex combination. Distributional assumptions for this model will be assessed using posterior predictive checks [27]. If a normal distribution is not suitable, alternative model functions, e.g., gamma and log-normal, will be considered. For IV ketamine, we will estimate the mean length of stay, onset of sedation, and duration of sedation by fitting a suitable distribution, chosen using posterior-predictive checks. The comparison of the means will then determine the probability that IN ketodex is superior to IV ketamine and declare significance using the algorithm outlined above.

To analyze the AEs, we will use a logistic dose-response model for IN ketodex and a binomial distribution for IV ketamine. We will then compute the posterior probability that optimal dose for IN ketodex has a lower AE rate than IV ketamine, declaring significance using the algorithm above. Each AE will be counted once for a given participant. We will also report the severity, frequency, and relationship of AEs to the study intervention by System Organ Class and preferred term groupings. For each AE, we will also report the start date, stop date, severity, relationship, expectedness, outcome, and duration. AEs leading to premature discontinuation from the study intervention and serious treatment-emergent AEs will be presented in either a table or a list.

##### Priors for secondary analyses

Priors for the model intercepts will be obtained from the literature, where possible, and down-weighted to reduce prior influence on the results. Priors for the regression coefficients will be central *t* distributions with precision of 0.001 and degrees of freedom 1 [25]. Priors for the standard deviations will use non-central *t* distributions (truncated at 0) [28]. We will ensure that these priors are vague with respect to the scale of the observed data and undertake sensitivity analyses to the prior specification.

#### Additional outcomes

We will report additional outcomes with descriptive statistics, using frequencies and percentages for categorical variables and means, medians, standard deviations, and interquartile ranges for continuous variables.

#### Additional analyses

We will use logistic regression to investigate the interaction between baseline pain, measured using the Faces Pain Scale - Revised, considered as a continuous variable, and treatment effect. The model will have a coefficient for treatment, baseline pain, and an interaction term between treatment and baseline pain. This analysis will only use data from the optimal dose combination. We will conclude a significant interaction effect if the 95% credible interval for the treatment interaction does not include zero.

## Trial status

The Ketodex study was registered on December 11, 2019, and started recruitment in March 2020. Recruitment is expected to be completed by December 2022. For the final analysis, the database will be cleaned and checked for completeness before being locked and the statistical analysis will then be undertaken using the methods in this SAP.

## Supplementary Information

**Additional file 1.**

## Data Availability

No datasets were used to develop this article as analysis was not undertaken. Thus, this consideration is not applicable.

## Abbreviations

AE: Adverse events
ALC: Average length criterion
DSMB: Data and safety monitoring board
ED: Emergency department
IN: Intranasal
IV: Intravenously
SAP: Statistical analysis plan
PSA: Procedural sedation and analgesia
PSSS: Pediatric Sedation State Scale
REB: Research Ethics Board
WCHRI: Women and Children’s Health Research Institute
