# Supplementary material for: The intranasal dexmedetomidine plus ketamine for procedural sedation in children, adaptive randomized controlled non-inferiority multicenter trial (Ketodex): a statistical analysis plan
Source: Trials. 2021 Jan 6;22:15. doi: 10.1186/s13063-020-04946-3 (PMC7789159; doi:10.1186/s13063-020-04946-3)
Supplement: Supplementary file 1 — Additional file 1. [file 13063_2020_4946_MOESM1_ESM.docx]

**Physician Surveys to Determine Non-Inferiority Margin for the Ketodex Trial**

To determine the non-inferiority margin for the Ketodex trial, the study team undertook two surveys of emergency department (ED) physicians. The first survey was distributed to ED physicians at the six participating sites for the Ketodex trial. The second survey was distributed to ED physicians at community hospitals around these participating sites. Surveys were sent to the hospital chiefs and disseminated by them using email to staff with the data collected in RedCAP.

The first survey elicited 94 responses and the second elicited 110 responses for a total of 204 physicians. The non-inferiority margin was taken as the mean of these 204 responses.

The survey to physicians at the six participating sites for the Ketodex trial used the following scenario:

Consider 100 five-year old children, all with a minimally angulated distal radius fracture. You believe that anatomical reduction will be achievable with no more than 10 minutes of manipulation. You have the option of providing either intranasal (IN) 10 mg/kg or intravenous (IV) ketamine 1.5 mg/kg. The dosing of both IV and IN ketamine is based on clinical practice guidelines and pilot work, respectively. Compared to IV, the IN route may offer the advantages of minimal distress for the child and caregiver, fewer nursing resources, lower cost, and greater caregiver satisfaction. Given these advantages and the fact that you can provide rescue sedation using IV ketamine if the IN route does not work, what is the largest number of children that fail IN ketamine sedation you would be willing to accept in order to routinely attempt IN ketamine as first line for sedation?

The survey to physicians at community hospitals used the following scenario:

Consider a five-year old child with a minimally angulated distal radius fracture in your E.D. You believe that you can easily reduce this with no more than 5 minutes of manipulation, and you have decided to use ketamine to sedate your patient. However, if you are unable to place an IV, you will need to transfer your patient to the academic paediatric E.D. in London. In order to avoid having to place an IV or transfer your patient, you have the option of using a new intranasal approach to giving ketamine, which is safe and well tolerated, but not effective in all patients i.e., some children will fail to develop sufficient sedation with intranasal ketamine administration. Out of a 100 similar children, what number of children would you be willing to accept failure of intranasal ketamine sedation and still try this approach as a first line option in an attempt to avoid having to place an IV or transfer your patient?

Survey Results**:**

| Non-inferiority Margin | 0 | 0.05 | 0.1 | 0.15 | 0.2 | 0.25 | 0.3 | 0.4 | 0.5 | 0.6 | 0.7 |
| --- | --- | --- | --- | --- | --- | --- | --- | --- | --- | --- | --- |
| Participating Site – Number of Responses | 0 | 22 | 40 | 11 | 12 | 9 | 0 | 0 | 0 | 0 | 0 |
| Community Hospitals – Number of Responses | 5 | 19 | 23 | 5 | 24 | 0 | 10 | 3 | 11 | 5 | 5 |
